# Activity of BET-proteolysis targeting chimeric (PROTAC) compounds in triple negative breast cancer

**DOI:** 10.1186/s13046-019-1387-5

**Published:** 2019-08-30

**Authors:** María del Mar Noblejas-López, Cristina Nieto-Jimenez, Miguel Burgos, Mónica Gómez-Juárez, Juan Carlos Montero, Azucena Esparís-Ogando, Atanasio Pandiella, Eva M. Galán-Moya, Alberto Ocaña

**Affiliations:** 1 Translational Research Unit, Albacete University Hospital, C/ Francisco Javier de Moya sn, 02006 Albacete, Spain; 2 Centro Regional de Investigaciones Biomédicas (CRIB), Universidad de Castilla-La Mancha (UCLM), C/Almansa 14, 02008 Albacete, Spain; 3 Instituto de Biología Molecular y Celular del Cáncer (IBMCC-CIC), Salamanca, Spain; 4 IBSAL, Salamanca, Spain; 5 CIBERONC, Salamanca, Spain; 6 CSIC, Salamanca, Spain; 7 Unidad de nuevas terapias y Oncología traslacional, Hospital Clínico Universitario San Carlos, IDISSC and CIBERONC, Calle del Prof Martín Lagos, s/n, 28040 Madrid, Spain

**Keywords:** PROTACs, BET inhibitors, Triple negative breast Cancer, Ovarian cancer, Resistance

## Abstract

**Background:**

Triple negative breast cancer (TNBC) is an incurable disease where novel therapeutic strategies are needed. Proteolysis targeting chimeric (PROTAC) are novel compounds that promote protein degradation by binding to an ubiquitin ligase. In this work, we explored the antitumoral activity of two novel BET-PROTACs, MZ1 and ARV-825, in TNBC, ovarian cancer and in a BET inhibitor resistant model.

**Methods:**

OVCAR3, SKOV3, BT549, MDA-MB-231 cell lines and the JQ1 resistant cell line MDA-MB-231R were evaluated. MTTs, colony-forming assay, three-dimensional cultures in matrigel, flow cytometry, and western blots were performed to explore the anti-proliferative effect and biochemical mechanism of action of MZ1 and ARV-825. In vivo studies included BALB/c *nu/nu* mice engrafted with MDA-MB-231R cells.

**Results:**

The BET-PROTACs MZ1 and ARV-825 efficiently downregulated the protein expression levels of the BET protein BRD4, in MDA-MB-231 and MDA-MB-231R. MZ1 and ARV-825 also showed an antiproliferative effect on sensitive and resistant cells. This effect was corroborated in other triple negative (BT549) and ovarian cancer (SKOV3, OVCAR3) cell lines. MZ1 provoked G2/M arrest in MDA-MB-231. In addition, a profound effect on caspase-dependent apoptosis was observed in both sensitive and resistant cells. No synergistic activity was observed when it was combined with docetaxel, cisplatin or olaparib. Finally, in vivo administration of MZ1 rescued tumor growth in a JQ1-resistant xenograft model, reducing the expression levels of BRD4.

**Conclusions:**

Using both in vitro and in vivo approaches, we describe the profound activity of BET-PROTACs in parental and BETi-resistant TNBC models. This data provides options for further clinical development of these agents in TNBC.

**Electronic supplementary material:**

The online version of this article (10.1186/s13046-019-1387-5) contains supplementary material, which is available to authorized users.

## Background

Triple negative breast cancer (TNBC) is a very aggressive tumor for which no curative therapies currently exists [1]. It accounts for around 15% of all breast tumors, and it is associated with poor prognosis, especially for patients with advanced disease, and a high grade of recurrence for those diagnosed in early stages [1, 2]. In this context, identification of oncogenic vulnerabilities that could be pharmacologically inhibited is a main goal.

Among the novel agents that have shown preclinical activity are those targeting the bromo and extra terminal domain proteins (BET) [3, 4], such as JQ1, currently in clinical development, which targets the BET protein BRD4. By inhibiting the acetylation of lysines of the tail of histones, BET inhibitors (BETi), such as the BRD4-targeting JQ1, reduce the expression of key oncogenic transcription factors, like DEP Domain Containing 1 (DEPDC), Forkhead box M1 (FOXM1), or LIM Domain Only 4 (LMO4), among others [5, 6]. However, as for most therapies, it is expected that resistance to these agents will eventually appear after a prolonged time of treatment, decreasing the therapeutic efficacy of these compounds. Moreover, several mechanisms have been described to be implicated in the resistance to this family of compounds, including the presence of a stem cell phenotype, the activation of polo-like kinase 1 (PLK1), or the basal activity of intracellular signaling kinases like protein kinase B (AKT) or Cyclin-Dependent Kinase Activating Kinase (CSK1) [7–12]. Reverting this resistance is crucial for BETi-based therapies to succeed.

Proteolysis targeting chimeric (PROTAC) molecules are a novel family of compounds with the ability to bind their target proteins and recruit an ubiquitin ligase, which promotes the targeted protein degradation [13]. In the case of BRD4-targeting agents, like the BET-PROTAC MZ1, leading to degradation of the target via the proteasome [14, 15]. This BET-PROTAC compounds have shown high activity in some hematological malignancies, like mantle lymphoma or acute myeloid leukemia (AML), compared with BETi [16], but no particularly result has been reported in breast cancer. Similarly, no evaluation of efficacy of these compounds has been reported in BETi resistant cells.

In the present study we aimed to explore if BET-PROTACs were able to revert resistance to BET inhibitors in a breast cancer model of TNBC. In addition, we explored their mechanism of action in sensitive and resistant cells. Our results show that BET-PROTACs are very active in both cell models, and are able to diminish tumor growth in an in vivo model of mice xenografted with cells resistant to BETi.

## Material and methods

### Cell lines culture and drugs

TNBC and ovarian cell lines, MDA-MB-231 and BT549 and SKOV3, respectively, were cultured in DMEM, and ovarian cells OVCAR3 were cultured in RPMI supplemented with inactivated fetal bovine serum (10%), antibiotics (100 U/mL penicillin and 100 /mL streptomycin) and L-glutamine (2 mM) (Gibco (Thermofisher), Sigma-Aldrich) (37 °C, 5% CO_2_). All cell lines used were provided by Drs. J. Losada and A. Balmain, who purchased them from the ATCC, in 2015. Cells authenticity was confirmed by STR analysis at the molecular biology unit at the Salamanca University Hospital. MDA-MB-231-derived resistant cell line (MDA-MB-231R) was obtained by pulsed exposure to increasing doses of JQ1 (72 h pulses every 2 weeks for 6 months).

BET inhibitors (JQ1 (HPLC: 99.6% purity) and OTX-015 (HPLC: 99.82% purity) and PROTACs-BRD4 (MZ1 (HPLC: 99.5% purity) and ARV-825 (LCMS: 99.37% purity)), together with the inactive form of MZ1, cis-MZ1 (HPLC: 98.6% purity), were purchase from Selleckchem (Houston, TX) and Tocris Bioscience (Bio-Techne R&D Systems, S.LU).

### MTT, colony formation, and 3D invasion assays

For MTT assay colorimetric assay, after treatments, cell medium was replaced with MTT solution (red phenol-free DMEM with MTT 0.5 μg/μL) (45 min, 37 °C). DMSO was then used to solubilize the samples. Absorbance values were recorded in a multiwell plate reader (555 nm with a reference wavelength of 690 nm). For synergy studies, we used the Chou-Talalay algorithm, which allows to obtain the combination index (CI) to determined which combinations were synergistic (CI < 1), additive (CI = 1), or antagonic (CI > 1) using Calcusyn 2.0 software.

For clonogenic assays, 24 h-treated cells were counted and seeded in triplicates for each condition. After 10 days, cells were fixed with glutaraldehyde (0.5%, 15 min) and, then, stained with crystal violet (0.05%, 15 min). Colonies were quantified using Image J software. For 3D invasion assays, cells were seeded on 48-wells plates containing a 1 mm layer of Matrigel (Sigma-Aldrich) and treated for 72 h. Matrigel generates a net that mimics the extracellular matrix. Invading 3D structures were evaluated using an inverted microscope and their diameter was quantified using Image J software.

### Flow cytometry experiments

For cell cycle analysis, after 12 h of treatment, cells were fixed in 70% ethanol in PBS (15 min). Cell pellets were washed in PBS + 2% BSA and incubated with Propidium iodide/RNAse staining solution (1 h, 4 °C, in dark; Immunostep).

For cell death studies, after 48 or 96 h of treatment, adherent and floating cells were collected and, after a wash with PBS, stained with Annexin Binding Buffer containing Annexin V-DT-634 and Propidium iodide (2 mg/mL) (1 h, RT, in dark; Immunostep). For caspase assays, cells were pre-treated with the pan-caspase inhibitor QVD (10 μM, 45 min; Sigma Aldrich) prior drug exposure.

All analyses were performed on a FACSCanto™ II flow cytometer using the FACS Diva software.

### Protein expression analysis: Western-blotting

For the evaluation of protein levels, MDA-MB-231 and MDA-MB-231R cells were seeded (500.000 cell/100 mm dish) and, the following day, treated sequentially: first, the 48 h points; the following day, the 24 h points; and finally, the 12 h points. All treatments were collected in parallel 72 h post-seeding together with their common non-treated control.

For the evaluation of cell cycle and apoptosis-related proteins, cells were treated for 12 h and 96 h, respectively. Then cells were lysed, and protein extracts (25–60 μg) were used for Western blot analyses with the indicated antibodies (Additional file 2).

### Caspase 3 activity

Caspase reaction buffer was added to the protein extract (50 μg, 1 h, 37 °C, in the dark). Then, fluorescence was measured (400/505 nm).

### In vivo studies

*BALB/c nu/nu* mice (4–5 weeks old, *n* = 13) mammary fat pads were injected with MDA-MB-231R (2.5 × 10^6^). Daily treatment with JQ1 (25 mg/kg, i.p.) was initiated when tumors reached a volume of 80–150 mm^3^. After 1 week of treatment with JQ1, a group of animals (*n* = 6) continued under this compound regime, while another group (*n* = 7) received a treatment of MZ1 (10 mg/kg, i.p.). Tumor growth was monitored for two more weeks. Then, tumors were collected, weighted, and stored at − 80 °C. For Western blot analysis, tumor samples (JQ1-treated *n* = 5; MZ1-treated *n* = 7) were homogenized with a sonicator Dispomix in ice-cold lysis buffer (1.5 mL/100 mg of sample). For protein levels evaluation, 60 μg of protein were used.

### Statistical analysis

We used t-test for independent samples non-parametric assay, together with the Levenne test to consider, or not, equal variances or ANOVA assay with Tukey subtype. The level of significance was considered 95% (* *p* ≤ 0.05; ** *p* ≤ 0.01 and *** *p* ≤ 0.001). Software GraphPad Prism and SPSS were used.

## Results

### PROTACs decrease BRD4 levels in BETi resistant cell lines

**Fig. 1:**
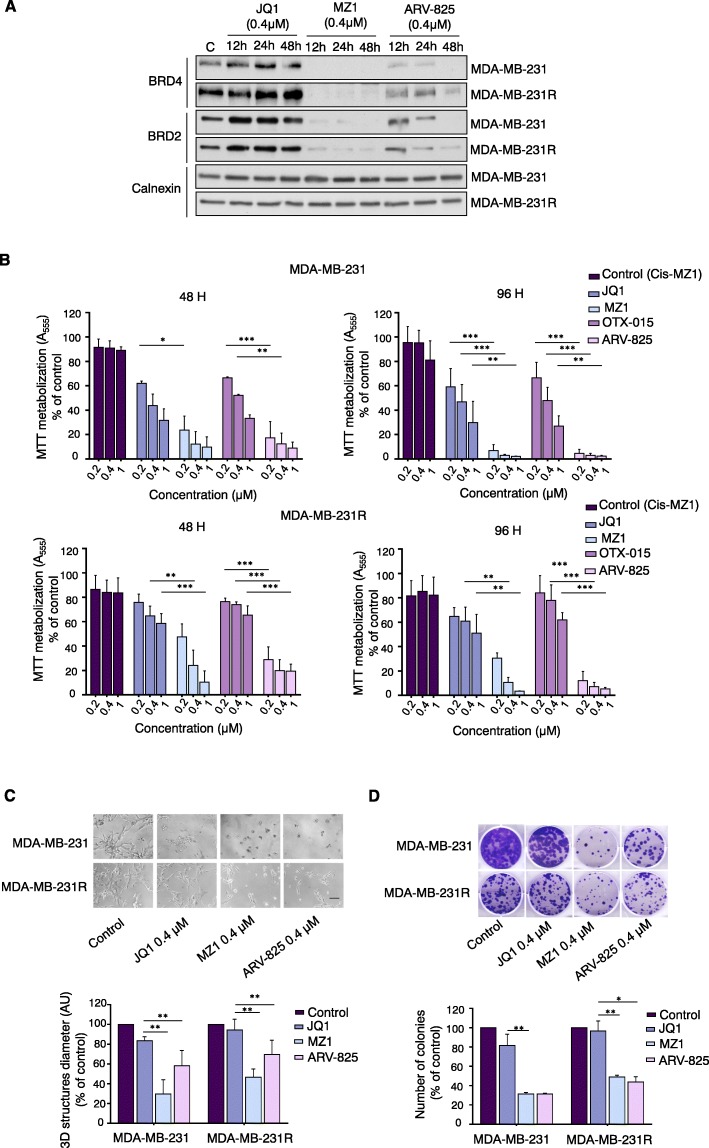
Evaluation of BRD4-PROTACs (MZ1 and ARV-825) efficacy in comparison with BET inhibitors (JQ1 and OTX-015) in MDA-MB-231 and MDA-MB-231-derived JQ1-resistant cells (MDA-MB-231R). **a**. MDA-MB-231 and MDA-MB-231R were treated with JQ1, MZ1, and ARV-825 for 12, 24, or 48 h (0.4 μM). Then, cells were lysed and 25 μg of total protein extract were analyzed by Western-blot with anti-BRD4 and anti-BRD2 antibodies. Calnexin was used as loading control.**b**.Parental and resistant-derived models were treated with JQ1, MZ1, OTX-015 and ARV-825 (0.2,0.4 and 1 μM). The inactive stereoisomer Cis-MZ1 was used as negative control. Cell viability was evaluated by metabolization of MTT after 48 or 96 h.**c**. MDA-MB-231 and MDA-MB231R were seeded in a semi-solid matrigel matrix the day prior the beginning of the treatments. Then, matrigel invasion capacity following a 72 h treatment with JQ1, MZ1, and ARV-825 (0.4 μM) was assessed and invading 3D structures were measured. Diameter scores are shown as arbitrary units. Scale bar = 100 μm. **d**. Colony formation ability after 12 h exposure to JQ1, MZ1, or ARV-825 (0.4 μM). Following the treatments, cells were seeded at low density (500 cells/well) and, 10 days later, fixed, stained with crystal violet, and counted. *p < 0.05; **p < 0.01; ***p < 0.001

First, we evaluated the BET-PROTAC inhibitor MZ1, based on the chemical structure of JQ1 [14], on parental MDA-MB-231 cells and on an exclusive model of JQ1-resistant TNBC cells derived from the above-mentioned. A profound downregulation of BRD4 and BRD2 was observed in both cell models upon MZ1 treatment, despite the fact that BRD4 basal expression levels were found to be much higher in the resistant model (Fig. 1a). Similarly, a reduction of the BET proteins levels was observed after ARV-825 treatment, a PROTAC that uses OTX-015, another BETi, as a backbone [15]. However, this effect was milder, particularly on BRD4 in the resistant model, what could be due to this elevated levels of this protein displayed by these cells (Fig. 1a).

### In vitro efficacy of BET-PROTACS in naïve and resistant cells

Next, we explored the antiproliferative effect of the BET-PROTAC inhibitors MZ1 and ARV-825 when compared with their counterparts JQ1 and OTX015, in MDA-MB-231 and MDA-MB-231R. Both BET-PROTACs induced a clear antiproliferative activity at different time points for both cell models, being this effect even greater in the resistant one (Fig. 1b). The effect of MZ1 and ARV-825 was further confirmed in matrigel invasion (Fig. 1c) and clonogenic assays (Fig. 1d), demonstrating a similar efficacy in both cell lines.

Given the activity of MZ1 and ARV-825 in naïve MDA-MB-231, we decided to explore their effect on other representative TNBC cell lines and extent this evaluation to ovarian cancer, given the molecular characteristics shared between both tumor types. A relevant antiproliferative activity of MZ1 and ARV-825 when compared with JQ1 and OTX-015 was observed in the TNBC cell line BT549 and in two ovarian cell lines, SKOV3 and OVCAR3 (Additional file 1: Figure. S1 A, B).

### Effect of PROTACs on cell cycle in JQ1 sensitive and resistant cells

**Fig. 2:**
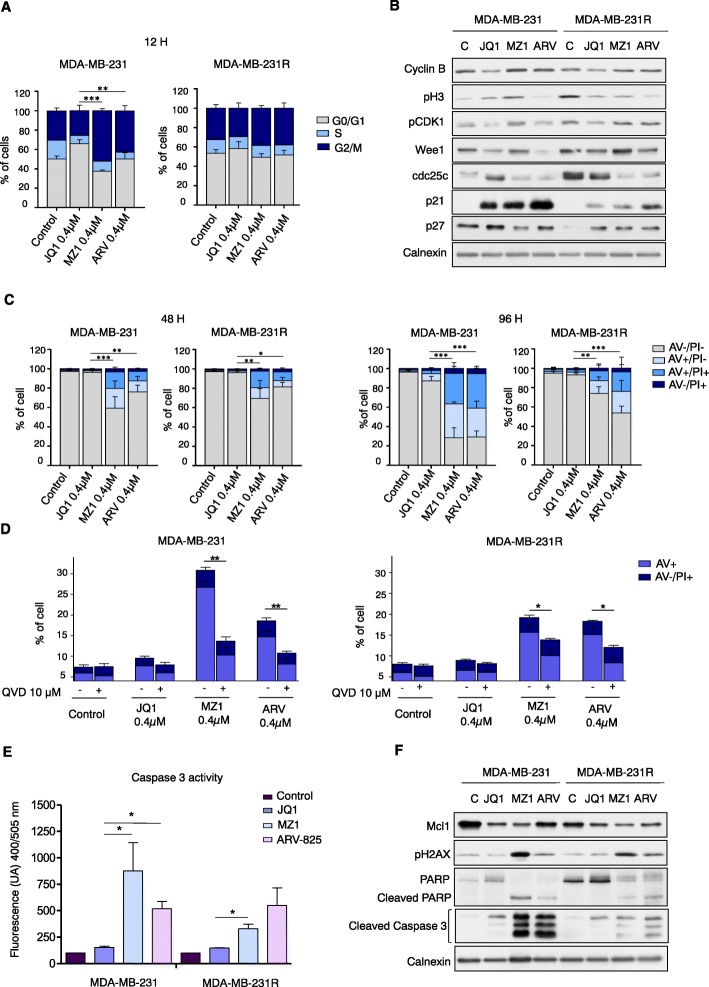
Cell cycle and cell death analyses in naïve and BETi-resistant MDA-MB-231 models. **a**. MDA-MB-231 and MDA-MB-231R cells were treated with JQ1, MZ1 and ARV-825 (0.4 μM) for 12 h. Then, cell cycle was evaluated by flow cytometry. Bar graphs show the percentage of cells in G0/G1, S, or G2/M cell cycle phases.**b**. MDA-MB-231 and MDA-MB-231R were treated with JQ1, MZ1, and ARV-825 for 12 h (0.4 μM). Then, cells were lysed and 50 μg of protein extract were analyzed by Western-blot with antibodies against proteins involved in cell cycle progression. Calnexin was used as loading control. **c**. Cell death produced by JQ1, MZ1 and ARV-825 (0.4 μM) in both cell lines was evaluated by flow cytometry with PI and Annexin V (AV) staining. Cells were classified in viable (AV -, PI -), early apoptotic (AV +, PI -), late apoptotic (AV +, PI +) and necrotic cells (AV -, PI+). **d**. MDA-MB-231 naïve and JQ1-resistant were pretreated with the pan-caspase inhibitor QVD (10 μM) for 45 min before being exposed to the drugs for 48 h. Cell death was analyzed by flow cytometry as described in C. **e**. Caspase 3 activity was measured by fluorescence (400/505 nm) and data were represented referred to control. *p < 0.05; **p < 0.01; ***p < 0.001. **f**. Cells were treated with JQ1, MZ1, and ARV-825 for 96 h (0.4 μM). Cells were then lysed and 50 μg of protein extract were analyzed by Western-blot with antibodies against proteins involved in apoptotic cell death. Calnexin was used as loading control

Bearing in mind that BET-PROTACs compounds showed a relevant antiproliferative effect in TNBC and ovarian cancer cell lines, and that these agents were able to overcome resistance to BETi, we decided to explore the molecular mechanism behind their activity in both naïve and resistant cell lines. To assess their impact on cell cycle, cells were first exposed to JQ1. This BETi was able to induce cell arrest in G1 in the sensitive cell line, while failed to activate this checkpoint in the resistant one (Fig. 2a). In contrast, MZ1 and ARV-825 increased G2/M in the sensitive model when compared with its resistant counterpart (Fig. 2a). Biochemical evaluation of cell cycle components showed that while PROTACs clearly augmented the expression of p21 in naïve cells, this increase was very slight in the resistant cells (Fig. 2b). Also, while JQ1 increased cdc25c levels in naïve MDA-MB-231, MZ1 and ARV-825 do not induce significant changes in this cyclin. Contrary, PROTACs decreased its levels in resistant cells, which actually exhibited higher basal levels of cdc25c, what correlates with a higher presence of resistant cells in G1. As expected, JQ1 do not strongly impact this cyclin in the resistant model (Fig. 2b). Globally, this data suggests that PROTACs mainly act on early mitosis arresting cells at the G2 phase.

### MZ1 and ARV-825 induce apoptosis in JQ1 resistant cells

We then explored the effect of PROTACs on cell death in both sensitive and resistant cells. Both MZ1 and ARV-825 were able to induce a marked increase in apoptosis at different time points, which was slightly higher in the sensitive model (Fig. 2c). Administration of a pan-caspase inhibitor reverted apoptosis in both models, suggesting that the mechanism was mainly caspase-dependent (Fig. 2d). In fact, the two PROTACs compounds activated caspase 3 in both cell lines, being this effect more clear in the naïve (Fig. 2e and f), as was also observed in the flow cytometry studies. This data support the effect observed after caspase inhibitor administration in this model. Biochemical evaluation of the mechanism of action showed that the levels of the antiapoptotic protein MCL1 were also reduced in both cells lines. Moreover, PROTACs were able to induce DNA damage by H2AX activation and PARP cleavage in both sensitive and resistant cell lines (Fig. 2f).

### Effect of MZ1 in combination with standard therapies

**Fig. 3:**
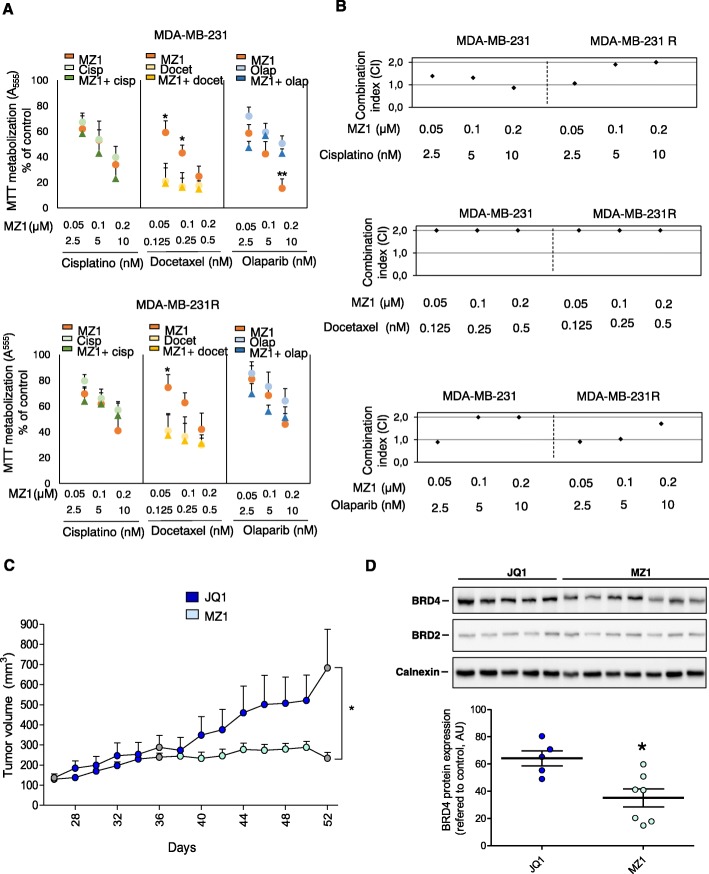
Effect of MZ1 in combination with standard therapies and in vivo efficacy of MZ1 in JQ1-resistant tumors. **a**. Antiproliferative properties of the combination of MZ1 and cisplatin, docetaxel, and olparib evaluated by MTT metabolization. Parental and resistant cells (MDA-MB-231) were treated with MZ1 (0.05, 0.1, and 0.2 μM), cisplatin (2.5, 5, and 10 nM), docetaxel (0.125, 0.25, and 0.5 nM), and olaparib (2.5, 5, and 10 nM) for 72 h as single agents or in combination. Statistics analysis was performed between MZ1 and combination results. **b**. Effect of MZ1 in combination treatments: synergy studies. Combination index (CI) for the different drug combinations were obtained using CalcuSyn program from viability values obtained in an MTT assay after 72 h of incubation with the drugs. Combination doses used are the same than in A. CI values lower than 0,8 indicate a synergistic action. **c**. Representation of the tumor volumes (mm^3^) of MDA-MB-231R-derived tumors treated with either JQ1 (25 mg/kg) for 3 weeks or MZ1 (10 mg/kg) for 2 week, after a 1 week treatment with JQ1. Tumors volumes were calculated as follow: V = (L× W^2^)/2, where V = volume (mm^3^), L = length (mm), and W = width (mm). Mean of tumor volume ± SEM was represented. *p < 0.05; **p < 0.01; ***p < 0.005. **d**. Expression levels of BRD4 and BRD2 in TNBC JQ1-resistant derived tumors. Tumor samples from C were collected, washed with cold PBS, minced, and homogenized in lysis buffer. Protein expression levels were analyzed by Western blotting as described above. Calnexin was used as a loading control. Image J software was used for quantification

Most therapies approved for solid tumors are based on combinations of anti-cancer agents [17]. In this context, we compared the anti-proliferative activity of MZ1 with other agents used in the clinical setting, including cisplatin, docetaxel, and the recently approved PARP inhibitor olaparib. As can be observed in Fig. 3a, MZ1 showed a significant antitumoral activity, being docetaxel the only agent that displayed a higher effect. This effect was similar in MDA-MB-231 and MDA-MB-231R. MZ1 combination with the mentioned therapies did not lead to a clear synergistic interaction for any combination in the either of the two cell models (Fig. 3b).

### In vivo efficacy of MZ1 in JQ1 resistant tumors

To explore the efficacy of MZ1 on JQ1-resistant tumors in vivo, MDA-MB-231R xenograft models were used. All animals were initially treated with JQ1 to ensure tumor resistance and, then, they were randomized into two groups. While tumors from animals that pursued JQ1 treatment continued growing, MZ1 prevented tumor progression in the other group (Fig. 3c). Evaluation of BRD4 levels in tumors lysates showed that average expression of this BET protein was lower in MZ1-treated mice when compared with mice treated with JQ1, confirming the effect of this BET-PROTAC in vivo. No such effect was observed for BRD2 (Fig. 3d).

## Discussion

In the present article we describe the anti-tumor activity and the mechanism of action of BET-PROTACs MZ1 and ARV-25 in TNBC and ovarian cancer cell lines, and in a JQ1-resistant TNBC cell line (MDA-MB-231R). At the present moment, information about the mechanism of action of this family of compounds in relation to BETi is limited to lymphoma and acute myeloid leukemia [16], and only limited data exists in solid tumors or in resistant models to BETi. Development of resistances is a relevant problem for all therapies after a prolonged treatment, and identification of agents that can act on that refractory population is a main objective with clear translation to the clinical setting.

In our study we observed a significant anti-tumoral activity of BET-PROTACs in TNBC and ovarian cancer, that was higher when compared to BETi that are currently in clinical development. This effect is observed using different approaches, including proliferation, invasion, and clonogenic assays. This data is in line with previous studies in AML and Lymphoma were these compounds showed potent lethality [16, 18].

BET-PROTACs were able to efficiently deplete BRD4 and BRD2 in both sensitive and resistant cell lines, being MZ1 more potent than ARV-825. Of note, the JQ1 resistant cell line showed higher basal levels of BRD4 when compared with its naïve counterpart. This finding is in line with reports that suggest that treatment with BETi do not downregulate the expression of BRD4 [18]. The efficient inhibition of BRD4 and BRD2 is translated to a significant induction of apoptosis in both sensitive and resistant cell lines. Notably, the mechanism mainly depended on caspases, as shown by the induction of caspase 3 and the inhibition of apoptosis observed upon treatment with a caspase inhibitor. In a similar manner, BET-PROTACs induced DNA damage, as measured by H2AX activation. Regarding the effect of these compounds on cell cycle, although BETi were able to induce arrest at G1, the effect of BET-PROTACs was more pleiotropic, showing a slight increase in G2/M. The increase expression of p21 and the reduction of cdc25c suggested an arrest at early G2 entry for both sensitive and resistant cells, results observed in other studies hematologic malignancies [18].

In comparison with agents used in the current clinical setting, MZ1 showed a relevant anti-proliferative activity, and only docetaxel displayed higher efficacy. MZ1 high activity is probably the reason for the lack of synergisms observed when combining MZ1 with chemotherapies. A comparable effect was observed with the approved PARP inhibitor olaparib. BET-PROTACs have shown synergistic interactions with bcl-2 and CDK4/6 inhibitors in lymphoma probably through the activation of compensatory pathways [16]. In addition, data suggest that PROTACS can revert resistant to current targeted therapies used in some hematological malignancies [16, 19].

Finally, animal studies confirmed the effect of MZ1 on the proliferation of JQ1-resistant tumors. We first confirmed that JQ1 resistant cells were also resistant when injected in nude mice. Next, we observed that administration of MZ1 reduced growth of these tumors in vivo*.* Evaluation of the resected tumors showed a reduction of BRD4 in MZ1-treated animals, confirming that the effect was secondary to the reduction of this protein. Conversely, no reduction of BRD2 was identified in contrast to the findings observed in cell lines, probably due to a milder effect of the compound on this protein.

## Conclusions

In this work we describe the efficacy of PROTACs in TNBC and ovarian cancer, and in a BETi-resistant TNBC model. Given the fact that BETi are currently in clinical development in TNBC and that therapeutic options available for this disease are limited, our findings provide evidence for the clinical development of these family of compounds for this indication.

## Additional files

Additional file 1: **Figure 1.** BET-PROTACs exercise more effect than BETi also in other triple-negative and ovarian models. TNBC cells (BT549) (A) and ovarian cells (SKOV3 and OVCAR3) (B) were treated with JQ1, MZ1, OTX-015, and ARV-825 (0.2,0.4, and 1 μM). The inactive stereoisomer Cis-MZ1 was used as negative control of treatment. After 48 or 96 h, viability cell was evaluated by metabolization of MTT. *p < 0.05; **p < 0.01; ***p < 0.001. (PDF 72 kb)

Additional file 2: **Table 1.** Reagents, instruments, sofwares, and buffers used in the study. (PDF 139 kb)

## Data Availability

The data that support the findings of this study are available from the corresponding authors upon reasonable request.

## Abbreviations

AKT: Protein kinase B
AML: Acute myeloid leukemia
BCA: Bicinchoninic acid
BET: Bromodomain Extra-terminal
BETi: Bromo and extraterminal domain inhibitors
BSA: Bovine Serum Albumin
CSK1: Cyclin-Dependent Kinase Activating Kinase
DEPDC: DEP Domain Containing 1
DMEM: Dulbecco’s Modified Eagle’s Medium
DMSO: Dimetilsulfoxide
FOXM1: Forkhead box M1
HPLC: High performance liquid chromatography
LCMS: Liquid chromatography-mass spectrometry
LMO4: LIM Domain Only 4
PBS: Phosphate Buffer Saline
PROTAC: Proteolysis targeting chimeric
TNBC: Triple negative breast cancer
