# Supplementary material for: Activity of BET-proteolysis targeting chimeric (PROTAC) compounds in triple negative breast cancer
Source: J Exp Clin Cancer Res. 2019 Aug 30;38:383. doi: 10.1186/s13046-019-1387-5 (PMC6717344; doi:10.1186/s13046-019-1387-5)
Supplement: Supplementary file 1 — Figure 1. BET-PROTACs exercise more effect than BETi also in other triple-negative and ovarian models. TNBC cells (BT549) (A) and ovarian cells (SKOV3 and OVCAR3) (B) were treated with JQ1, MZ1, OTX-015, and ARV-825 (0.2,0.4, and 1 μM). The inactive stereoisomer Cis-MZ1 was used as negative control of treatment. After 48 or 96 h, viability cell was evaluated by metabolization of MTT. *p < 0.05; **p < 0.01; ***p < 0.001. (PDF 72 kb) [file 13046_2019_1387_MOESM1_ESM.pdf]

Reagent	Obtained by:
Dulbecco's Modified Eagle's Medium (DMEM)	Gibco (Thermofisher)
Roswell Park Memorial Institute's Medium (RPMI)	Sigma-Aldrich
JQ1 and OTX-025 (BET inhibitors)	Selleckchem (Houston, TX)
MZ1 and ARV-825 (PROTACs-BRD4)	Tocris Bioscience (Bio-Techne R&D Systems, S.LU).
Dimetilsulfoxide (DMSO)	Fisher-Scientifics
Matrigel	Sigma-Aldrich
Propidium iodide/RNAse staining solution	Immunostep S.L., Salamanca, Spain
Annexin V-DT-634	Immunostep S.L., Salamanca, Spain
Bicinchoninic acid (BCA) protein assay kit	Sigma-Aldrich
Polyvinylidene difluoride membranes	Millipore Corporation, Bedford, MA
Anti-cyclin B, anti-PARP, anti-Mcl1	Santa Cruz Biotechnology, USA
Anti-p(Y15) CDK1, anti-p27, anti-pH2AX, anti-Wee1, anti-p21, anti-BRD2, anti-BRD4, anti-cleaved caspase 3	Cell Signalling Technologies, Beverly, MA, USA
Anti-pHistone H3	Millipore Corporation
Anti-Calnexin	Stressgen Bioreagents, Canada

Instrument or software	Obtained by:
ECL Plus Western Blotting Detection System	GE Healthcare, Buckinghamshire, United Kingdom
Calcusyn 2.0 software	Biosoft, Ferguson, MO
Dispomix	L&M Biotech, Holly Springs, NC, USA
FACSCanto ™ II flow cytometer	BDBiosciences
Multiwell plate reader	BMG labtech
SPSS version 22	IBM SPSS Statistics
GraphPad Prism version 5	GraphPad Software

Buffer	Compounds
Annexin binding buffer	10 mM HEPES, pH 7.4, 140 mM NaOH, 2.5 mM CaCl2
Caspase reaction buffer	50 mmol/L HEPES pH 7.4, 300 mmol/L NaCl, 2 mmol/L EDTA, 0.2% CHAPS, 20% sucrose, 20 mmol/L ditritiol (DTT) and 10 mmol/L substrate caspase Ac-DEVD-AFC
Tris-buffered saline with Tween (TBST)	100 mM Tris [pH 7.5], 150 mM NaCl, 0.05% Tween 20
Lysis buffer	20 mM Tris–HCl [pH 7.0], 140 mM NaCl, 50 mM EDTA, 10% glycerol, 1% Nonidet P-40, 1 µM pepstatin, 1 µg/mL aprotinin, 1 µg/mL leupeptin, 1 mM phenylmethyl sulfonyl fluoride, 1 mM sodium orthovanadate
