# Supplementary figures and images for: Activity of BET-proteolysis targeting chimeric (PROTAC) compounds in triple negative breast cancer
Source: J Exp Clin Cancer Res. 2019 Aug 30;38:383. doi: 10.1186/s13046-019-1387-5 (PMC6717344; doi:10.1186/s13046-019-1387-5)

**A**

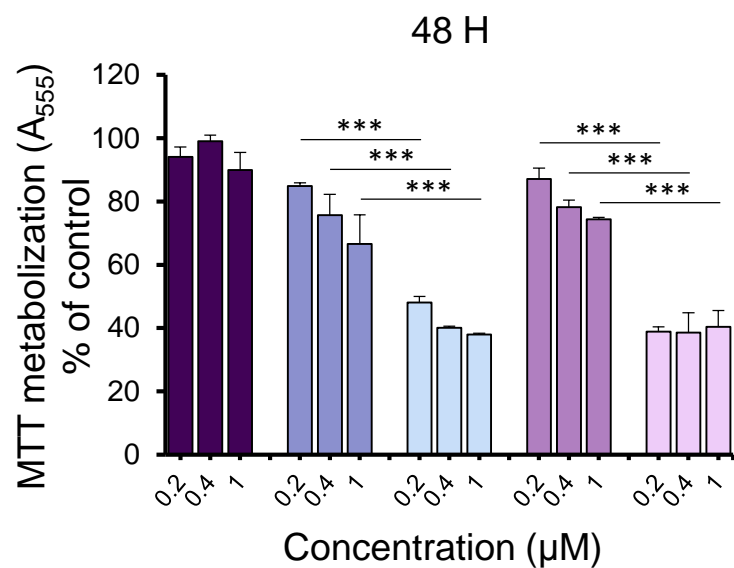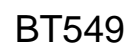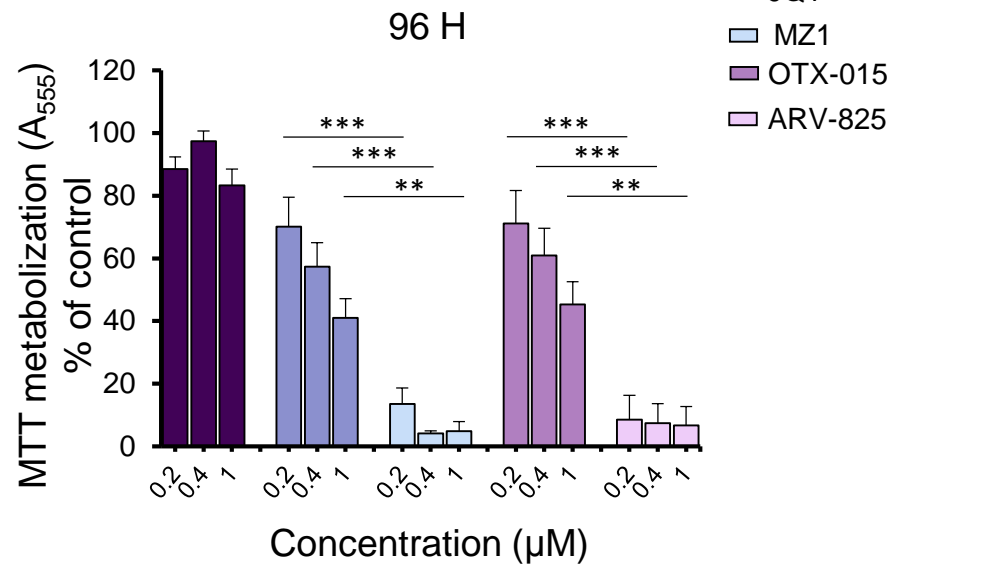

# B

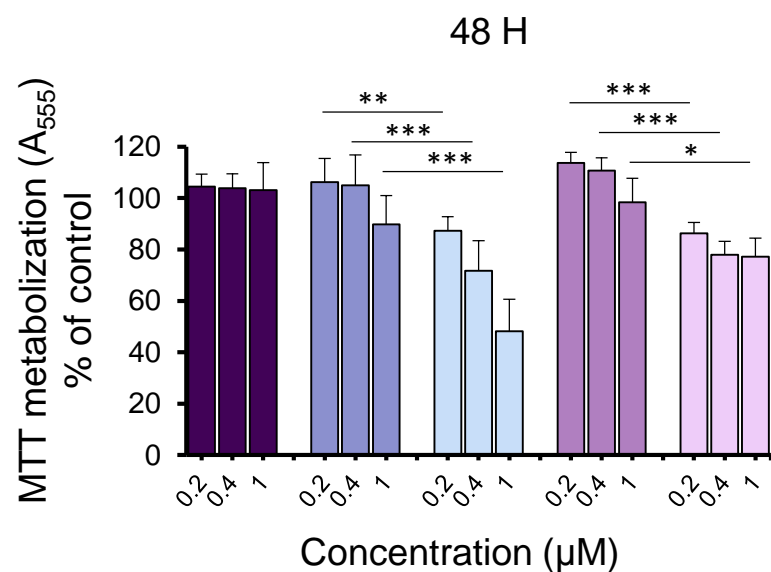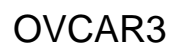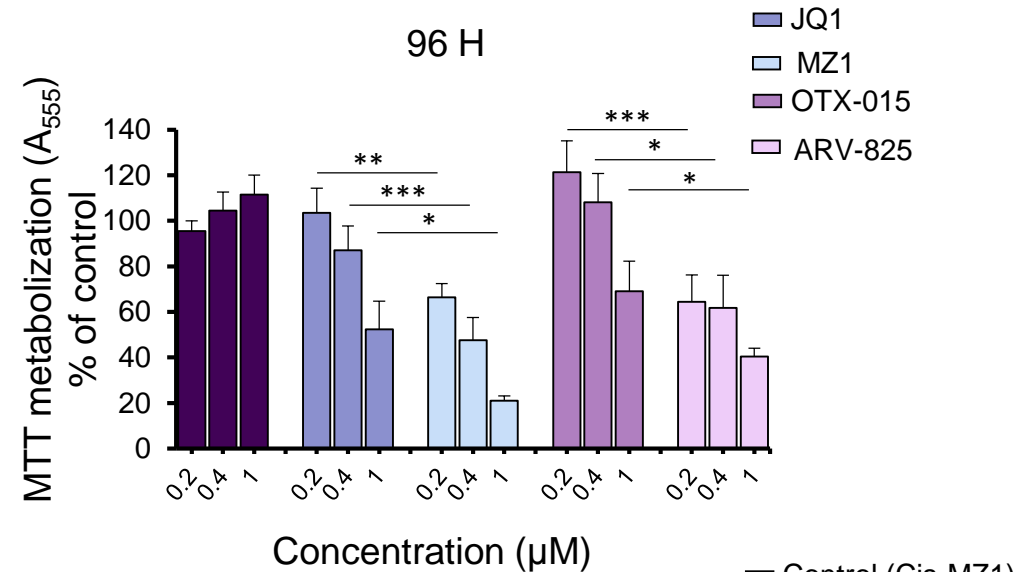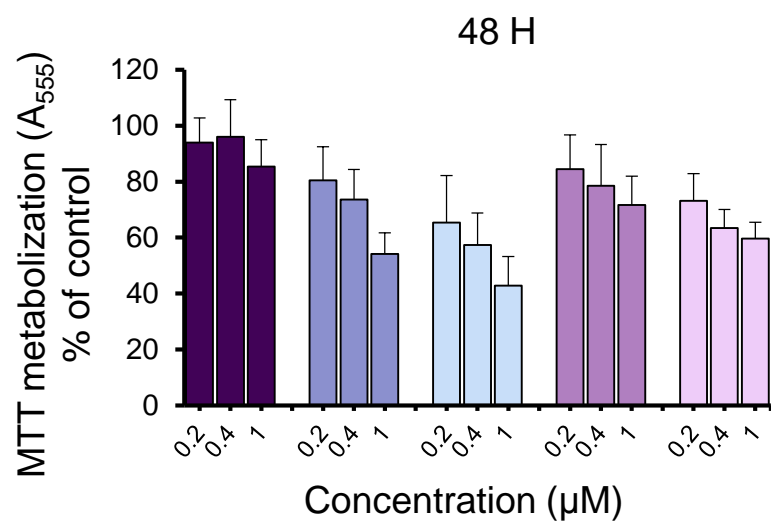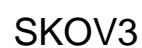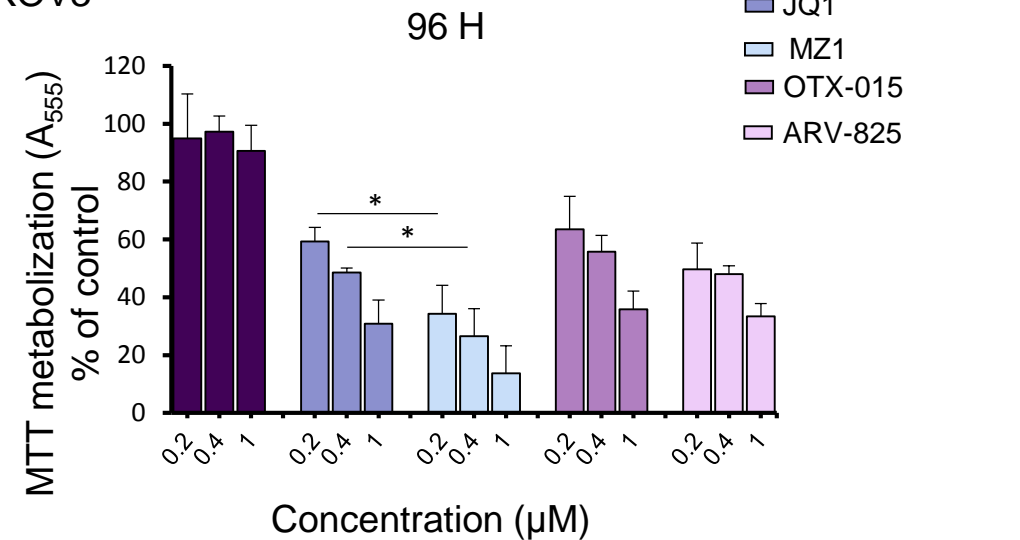

## Supplementary figure 1

Supplement: Supplementary file 2 — Table 1. Reagents, instruments, sofwares, and buffers used in the study. (PDF 139 kb) [file 13046_2019_1387_MOESM2_ESM.pdf]
